# Physically Transient, Flexible, and Resistive Random Access Memory Based on Silver Ions and Egg Albumen Composites

**DOI:** 10.3390/nano12173061

**Published:** 2022-09-03

**Authors:** Lu Wang, Yukai Zhang, Peng Zhang, Dianzhong Wen

**Affiliations:** School of Electronic Engineering, Heilongjiang University and Heilongjiang Provincial Key Laboratory of Micro-Nano Sensitive Devices and Systems, Heilongjiang University, Harbin 150080, China

**Keywords:** physically transient, resistive memory, silver ions, egg albumen, logic gate

## Abstract

Organic-resistance random access memory has high application potential in the field of next-generation green nonvolatile memory. Because of their biocompatibility and environmental friendliness, natural biomaterials are suitable for the fabrication of biodegradable and physically transient resistive switching memory devices. A flexible memory device with physically transient properties was fabricated with silver ions and egg albumen composites as active layers, which exhibited characteristics of write-once-read-many-times (WORM), and the incorporation of silver ions improved the ON/OFF current ratio of the device. The device can not only complete the logical operations of “AND gate” and “OR gate”, but its active layer film can also be dissolved in deionized water, indicating that it has the characteristics of physical transients. This biocompatible memory device is a strong candidate for a memory element for the construction of transient electronic systems.

## 1. Introduction

Currently, the speed of the updating and iteration of consumer electronic products is increasing, but these devices often have problems that cannot be degraded, and eventually become electronic waste. Whether through burial or burning, it will place great pressure on the ecological environment [1]. Since biomaterials have the advantages of biocompatibility, degradability, low cost, light weight, high flexibility, and diverse functions, they have shown application potential in biomedicine and wearable electronic devices [2,3,4]. Therefore, electronic devices prepared by biomaterials have attracted increasing attention [5,6,7]. On the other hand, the increasing demand for green storage devices in the future has promoted the development of storage technology based on natural biomaterials. Among the emerging nonvolatile memories, resistive random access memory (RRAM) has the advantages of high speed and low power consumption and has received close attention by researchers [8,9,10,11,12,13,14]. At present, chitosan [15,16], ferritin [17,18], DNA [19,20], sericin [21,22], gelatin, and other biomaterials have been widely used in research on bio-resistive memory devices [23,24,25].

Physically transient resistance random access memory can disappear under special conditions, which makes the device suitable for bioelectronics and security storage devices [26,27,28,29]. Currently, transient electronic devices based on natural biomaterials have attracted great research interest. A storage device was prepared using water-soluble solid glycerol nanopaper as a substrate, and the device could be degraded when placed in water [30]. A resistive switching memory device was fabricated on an ITO electrode using α-lactose as the dielectric layer material. The device, using the silver top electrode, could be dissolved in deionized water and exhibited physically transient properties [28]. The W/silk fibroin/Mg memory device was prepared by introducing silk fibroin as the active layer. The device was dissolved in phosphate buffer and exhibited physically transient properties [31]. A memory device with the structure of Al/gelatin/Ag was fabricated on the biocellulose membrane, and the cellulose membrane could be completely degraded in soil after 5 days. It can be seen that biomaterials have the potential to prepare novel physically transient devices, which can partially or completely disappear physically when the physically transient devices are stimulated externally. This new type of physically transient device can meet the needs of future transient information storage devices [32].

In this work, a flexible, transparent, biocompatible physically transient memory device with a sandwich structure was fabricated by using egg albumen (EA) doped with a silver ion composite (EA:Ag^+^) as the active layer, which exhibited stable WORM properties. The electrical properties of the device under different bending radii were tested, demonstrating the stability of the device and its potential for flexible applications. By doping soluble Ag^+^, the ON/OFF current ratio of the device is significantly improved, and the false reading rate of the device in circuit applications is greatly reduced. The logic operation of “AND gate” and “OR gate” was realized by this device. Interestingly, the active layer film could be completely dissolved in deionized water within 30 min, accompanied by the disappearance of resistive state switching behavior. Therefore, flexible physically transient memory devices based on silver ions and egg albumen composites have broad prospects for green and safe storage.

## 2. Materials and Methods

### 2.1. Preparation of the Device

A sandwich-structured Al/EA:Ag^+^/ITO memory device was fabricated on a flexible PET substrate. The ITO/PET transparent substrate (the thickness of substrate is 0.175 mm) was cleaned prior to device fabrication. The ITO/PET was placed in acetone, absolute ethanol, and deionized water, and ultrasonically cleaned for 15 min each time. The egg albumen solution and deionized water (DI) were diluted at a volume ratio of EA:DI = 1:10 and sonicated for 15 min. The diluted egg albumen was mixed with 1 mmol/L silver nitrate solution, and sonication was continued for 15 min to obtain a silver-ion-doped egg albumen solution. Next, the diluted EA solution (for undoped devices) and the silver ion-doped egg albumen solution (for doped devices) were spin-coated at 500 rpm for 5 s and 4000 rpm for 40 s on the ITO/PET substrate, respectively. The samples were dried at 80 °C for 10 min. Finally, the top aluminum electrode was formed by thermal evaporation under a vacuum of 2 × 10^−3^ Pa, and the preparation of Al/EA/ITO and Al/EA:Ag^+^/ITO RRAM was completed.

### 2.2. Feature Description

The UV-Vis spectra of EA were obtained using a UV-Vis spectrophotometer (UV/VIS, TU-1901). The electrical properties of the prepared memory device were tested using a semiconductor parametric tester (Keithley 4200).

## 3. Results

### 3.1. Device Structure and Material Characterization

**Figure 1:**
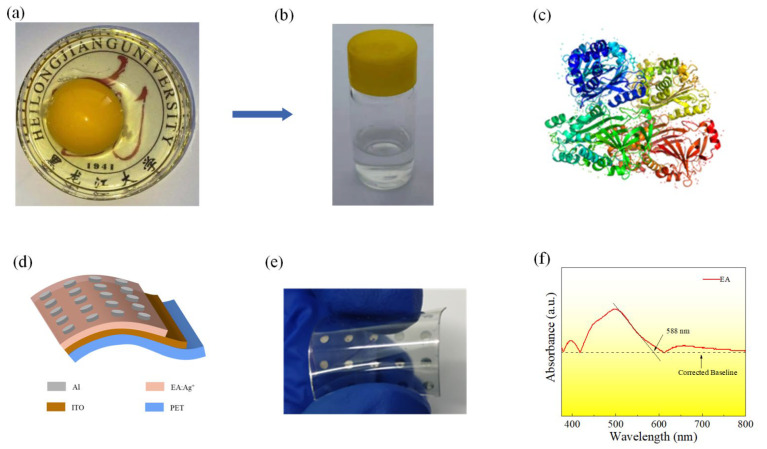
(**a**) The egg white and yolk of a whole egg. (**b**) Silver-ion-doped egg albumen solution. (**c**) Three-dimensional schematic diagram of ovalbumin. (**d**) Schematic diagram of Al/EA:Ag^+^/ITO memory devices. (**e**) Physical image of Al/EA:Ag^+^/ITO memory devices. (**f**) UV-Vis spectra of the EA film.

Figure 1a shows the whole egg composed of the egg white and yolk, using egg albumen as the active layer material. Figure 1b shows EA doped with silver ions. Ovalbumin accounts for approximately 54–69% of egg albumen protein. Figure 1c shows the 3D structure of ovalbumin in the egg albumen. Figure 1d,e are schematic and physical images of bendable and transparent flexible devices, respectively. As shown in Figure 1f, the EA film was analyzed by UV-Vis spectroscopy. Eg is the point of intersection of the edge tangent through the absorption peak with the corrected baseline, and the edge of the absorption peak of the EA film is calculated at 588 nm. From the relation:

E_g_ = hc/λ (1)

the optical band gap of the material can be calculated as 2.11 eV.

### 3.2. Memristor Performance

**Figure 2:**
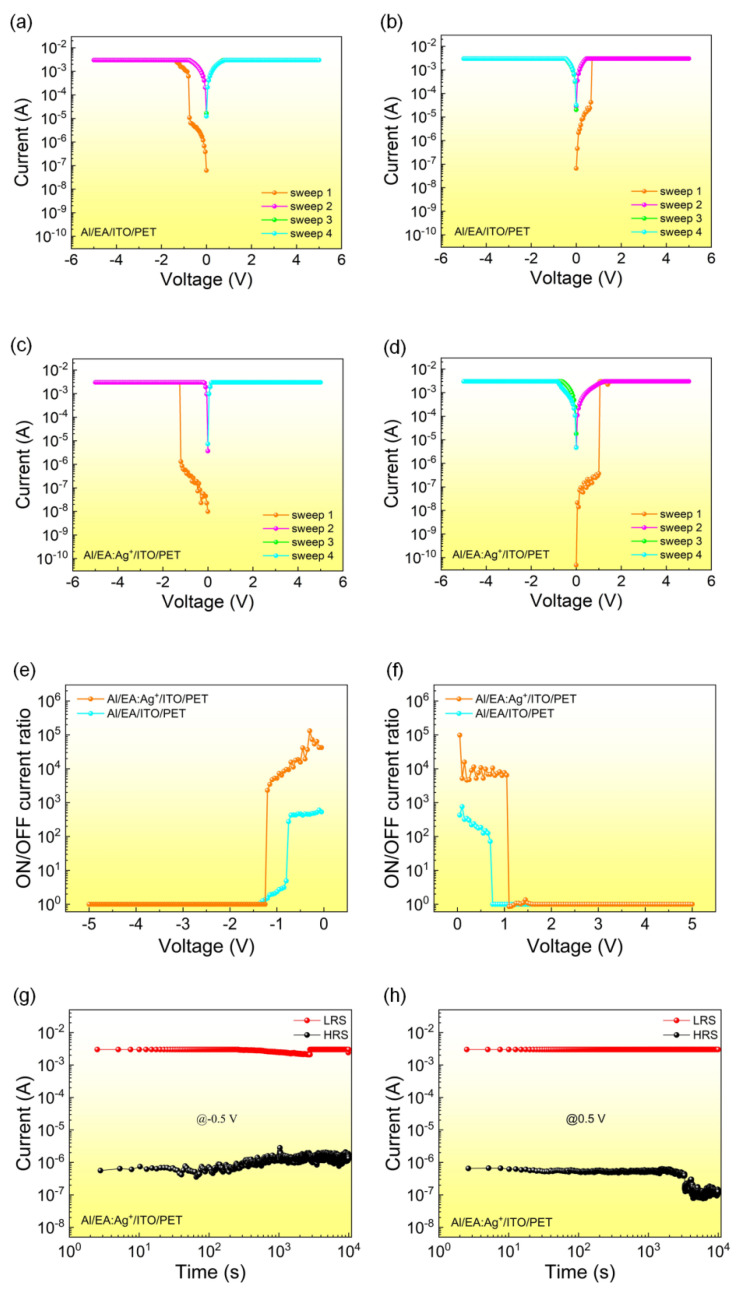
Current–voltage characteristic curve of the Al/EA/ITO device: (**a**) initial application of negative voltage and (**b**) initial application of positive voltage. *I–V* characteristic curve of the Al/EA:Ag^+^/ITO device: (**c**) initial application of negative voltage and (**d**) initial application of positive voltage. ON/OFF current ratio of the Al/EA:Ag^+^/ITO device: (**e**) initial application of negative voltage and (**f**) initial application of positive voltage. Retention time of the Al/EA:Ag^+^/ITO device: (**g**) initial application of negative voltage (read voltage is −0.5 V) and (**h**) initial application of positive voltage (read voltage is 0.5 V).

Figure 2a,b shows the typical *I–V* characteristic curves of the Al/EA/ITO device. The initial state of the Al/EA/ITO device is a high resistance state (HRS). When the voltage reaches a threshold voltage (V_th_) of −0.75 V, the state of the device switches to a low resistance state (LRS). In the next three tests, the LRS is still maintained, indicating that the device has a WORM feature. Likewise, the tested *I–V* curves of the Al/EA:Ag^+^/ITO memory device are shown in Figure 2c,d. The device still has the same characteristics. As shown in Figure 2e,f, the average ON/OFF current ratio (2.00 × 10^5^) of Al/EA:Ag^+^/ITO is improved compared with that of Al/EA/ITO (3.63 × 10^2^) by three orders of magnitude. The device has a low false-read rate in the circuit. The test results of the Al/EA:Ag^+^/ITO device’s retention ability are shown in Figure 2g,h. The current values corresponding to the HRS and LRS of the device were recorded at −0.5 V and 0.5 V. This resistance state remains stable for more than 10^4^ s. Therefore, the retention ability of the flexible device Al/EA:Ag^+^/ITO is proven to be reliable.

**Figure 3:**
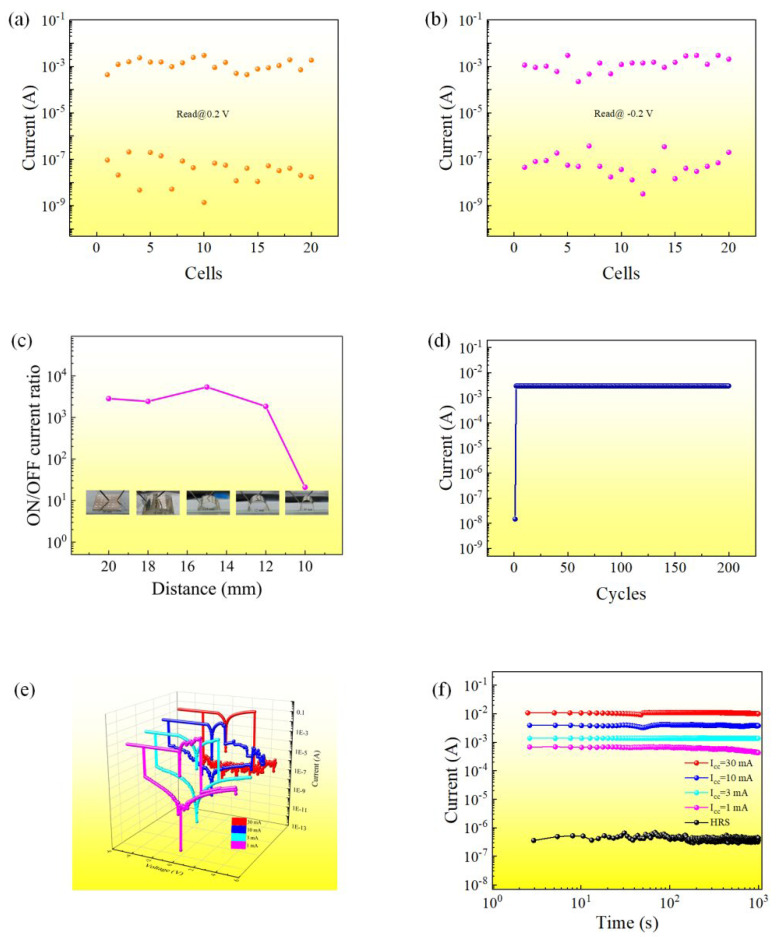
Current value for HRS and LRS of 20 cells of Al/EA:Ag^+^/ITO (**a**) read voltage is −0.2 V (**b**) read voltage is 0.2 V. (**c**) Bending stability of the Al/EA:Ag^+^/ITO device. (**d**) Switching of the Al/EA:Ag^+^/ITO device cycle; the current value is read at −0.5 V. Multilevel test results of the Al/EA:Ag^+^/ITO device: (**e**) typical *I–V* characteristic curve and (**f**) retention time.

The yield of the Al/EA:Ag^+^/ITO device was determined. The electrical characteristics of the 20 cells of the device were tested. The resistance values of the HRS and LRS of the device were read at 0.20 V and −0.20 V, respectively, as shown in Figure 3a,b.

The overall ON/OFF current ratio of the device is still significantly improved compared with that of the device without silver ions. We tested the mechanical flexibility of the memory cell, as shown in Figure 3c. When the flexible Al/EA:Ag^+^/ITO device (20 mm × 20 mm in size) was gradually bent from a flat state to a diameter of 12 mm, the device could still work normally and exhibited the characteristics of WORM. When the bending distance was further increased to 10 mm, the device was damaged. The results show that the device has excellent bending stability over a wide range. Figure 3d shows the cyclic endurance test result of the Al/EA:Ag^+^/ITO device. At 200 cycles, the resistance value in the ON state did not change significantly, proving that the device has good endurance.

When a negative compliance current (*I*_CC_^−^) of 1 mA is applied at a negative voltage, and a positive compliance current (I_CC_^+^) of 100 mA is applied at a positive voltage, the *I–V* characteristic curve of the device exhibits bistable switching behavior. The current–voltage characteristic curves shown in Figure 3e are about the negative voltage applied with different compliance currents (I_CC_^−^ = 1 mA, I_CC_^−^ = 3 mA, I_CC_^−^ = 10 mA, I_CC_^−^ = 30 mA) and under the positive voltage I_CC_^+^ = 100 mA. When the compliance current gets higher, the LRS resistance of the device decreases, and the HRS resistance is almost unchanged, which proves that the device has multilevel memory capability. As shown in Figure 3f, the retention time test was carried out for the multilevel state of the device. When a sustained voltage of −0.50 V is applied to the device, the high and low resistance states of the device remain stable for more than 10^3^ s. This demonstrates the multilevel state storage potential of egg-albumen-based bio-RRAM doped with silver ions.

**Figure 4:**
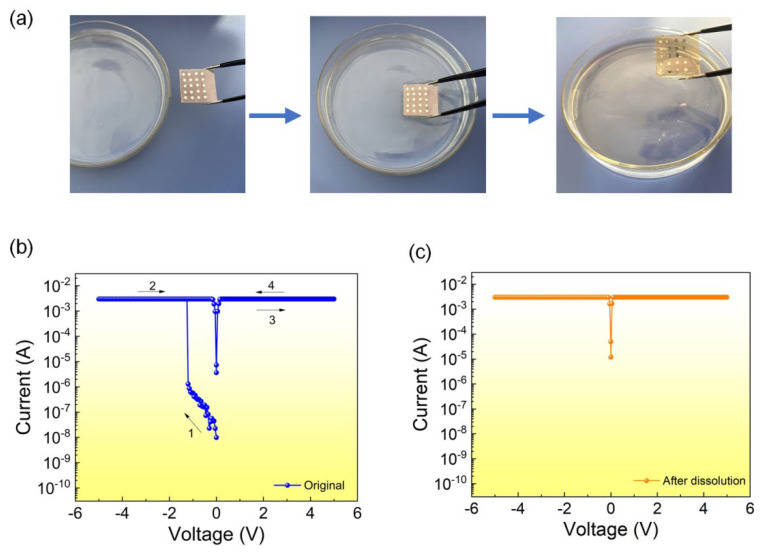
(**a**) The EA:Ag^+^ films after dissolution in DI water. (**b**,**c**) The electrical properties of the original Al/EA:Ag+/ITO device and after dissolution.

Figure 4a shows a series of pictures of the dissolution of the active layer of the device. After soaking for 30 min, the active layer’s thin film disappeared. The electrical properties of the Al/EA:Ag^+^/ITO device were further tested. The electrical characteristics of the device disappear, and the resistance state switching behavior cannot be accomplished. The results show that memory devices based on silver-ion-doped egg albumen films have application potential as physically transient electronic devices. It can also serve as a green and secure data storage device.

**Figure 5:**
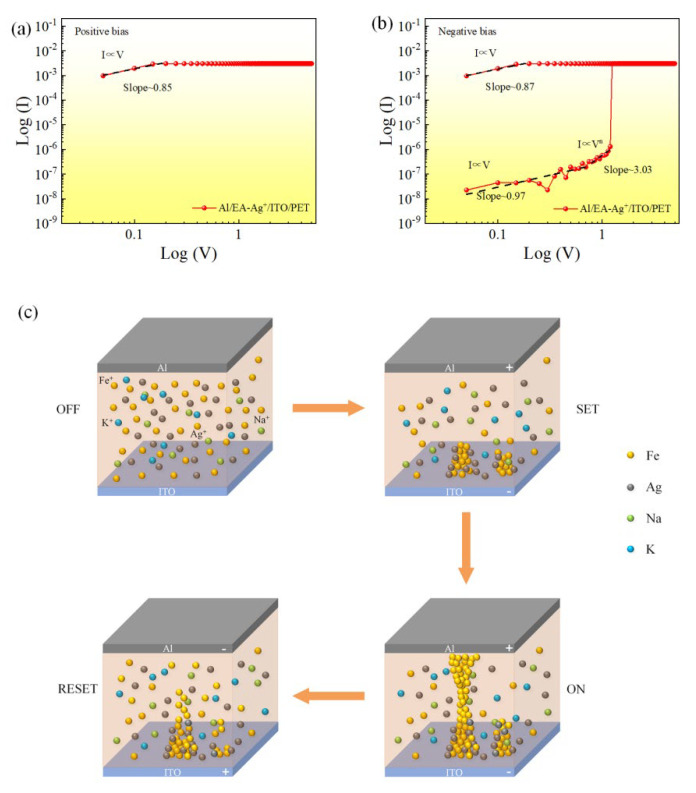
(**a**,**b**) *I–V* curves on a log–log scale for the Al/EA:Ag^+^/ITO structure. The scatters are experimental data, and the straight lines are the fitting curves from theoretical models. (**c**) Diagram of the conduction mechanism.

To further explore the internal current transport mechanism of the flexible Al/EA:Ag^+^/ITO device, as shown in Figure 5a,b, the *I–V* characteristic curves were plotted and fitted on a log–log scale. At low voltages, the slope value is approximately 1. The voltage continues to increase, and the slope satisfies the relation *I∝V*^2^. In the high-voltage region, the slope satisfies *I∝V*^3^, indicating that the current transport mechanism of the device is dominated by the space-charge-limited current (SCLC) mechanism [33,34,35]. The conductivity scheme of the Al/EA:Ag^+^/ITO device is illustrated in Figure 5c. When no external voltage is applied, the initial state of the device is the HRS, and the distribution of particles in the active layer is random. When a positive voltage is applied to the top electrode of the device, and the bottom electrode of the device remains grounded, the metal ions move toward the bottom electrode under the action of the electric field. At the same time, the electrons injected from the bottom electrode reduce the iron ions and silver ions in the active layer to iron atoms and silver atoms, respectively. Conductive filaments are formed, and the device is in the LRS. When a negative bias is applied to the top electrode of the device, the reverse process occurs. The iron and silver atoms accumulated near the top electrode are oxidized to an ionic form, causing the conductive filament to break and the device to switch to HRS. The device exhibits bipolar resistive switching behavior.

Due to the Coulomb blocking effect of silver nanoparticles, once an electron enters the nanoparticle, it will prevent other electrons from entering the nanoparticle. The HRS resistance of the device increases, and the ON/OFF current ratio increases. When a compliance current of 3 mA is applied in both the positive and negative directions, the external energy after the formation of the conductive filaments is insufficient to break the conductive filaments. At this time, the device exhibits the characteristics of WORM.

**Figure 6:**
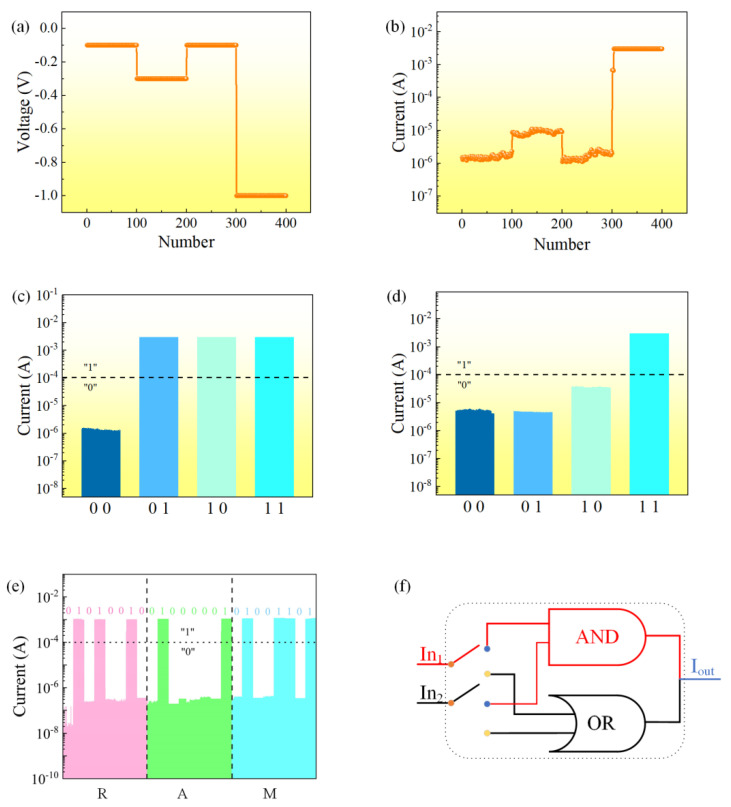
Al/EA:Ag^+^/ITO device: (**a**) input voltage and (**b**) current response. (**c**) Logic “OR gate”. (**d**) Logic “AND gate”. (**e**) Character display. (**f**) Working principle.

The response of device resistance switching to voltage was tested, and voltages of −0.10 V, −0.30 V, −1 V, and −0.10 V were applied to the device under the condition of I_CC_ = 3 mA, as shown in Figure 6a. At a read voltage of −0.10 V, it can be seen that the initial state of the device has not changed and is still in the HRS. Applying −1 V to the device switches the device state to LRS, as shown in Figure 6b.

The logical function of each individual component is determined by the configured hardware. Field programmability depends heavily on the performance of the logical storage device. The resistive state of the Al/EA:Ag^+^/ITO device is dependent on the externally applied voltage. Therefore, the resistive state of the device can be obtained using the external input voltage as an independent input. Independent electrical pulses are applied to control the output signal.

When the response current exceeds 10^−4^ A, the logic state is defined as “1”. When the current is less than 10^−4^ A, the logic state is defined as “0”. When a single or simultaneous input of two external input signals of −1.2 V is applied to the device, the current exceeds 10^−4^ A at this time, so logic “1” can be represented by a single input or a simultaneous input of a −1.20 V voltage signal. This is equivalent to the logic gate “OR gate”, as shown in Figure 6c.

An equally important gate circuit is the “AND gate”, as shown in Figure 6d. When a voltage of −0.60 V is used as the input signal, the output current depends on the input voltage signal. With only two input signals simultaneously at −0.60 V, the output current can reach 10^−4^ A. That is, by applying two high levels to the device at the same time, the logic state “1” can be achieved; otherwise, the logic state is “0”, equivalent to the “AND gate” in the logic gate circuit. Figure 6f shows a working schematic of the schematic logic unit.

The response of the current to the input signal shows that the output current can be well maintained when different input signals are applied to the device (Figure 6b). According to ASCII, the letter “R” can be represented by the code “01010010” (Figure 6e). The other letters “A” and “M” can be displayed in a similar manner. RAM, short for random access memory, can be demonstrated with 24 Al/EA:Ag^+^/ITO cells. Therefore, the device completes the function of displaying characters by applying external input signals.

## 4. Conclusions

In summary, Al/EA:Ag^+^/ITO memory devices with physically transient properties were fabricated, which exhibited the electrical properties of WORM. Compared with the pure albumen device, the ON/OFF current ratio of the device doped with silver ions is greatly improved (the average ON/OFF current ratio is increased from 3.63 × 10^2^ to 2.00 × 10^5^). The storage characteristics of the device in the bent state are the same as those in the flat state, and the device has good bending stability. Utilize the device to complete the character display function and implement the “AND” and “OR” logic functions. The active layer of the device can be dissolved in deionized water, indicating that the device has controllable transient behavior and great potential for biomedical electronics applications.

## Data Availability

Not applicable.

## References

[1] Zoeteman B.C.J., Krikke H.R., Venselaar J. (2009). Handling WEEE waste flows: On the effectiveness of producer responsibility in a globalizing world. Int. J. Adv. Manuf. Technol..

[2] Mukherjee C., Hota M.K., Naskar D., Kundu S.C., Maiti C.K. (2013). Resistive switching in natural silk fibroin protein-based bio-memristors. Phys. Status Solidi (a).

[3] Ouyang J., Chu C.W., Szmanda C.R., Ma L., Yang Y. (2004). Programmable polymer thin film and non-volatile memory device. Nat. Mater..

[4] Wang L., Yang T., Wen D. (2021). Tunable Multilevel Data Storage Bioresistive Random Access Memory Device Based on Egg Albumen and Carbon Nanotubes. Nanomaterials.

[5] Zhu Z., Kin Tam T., Sun F., You C., Percival Zhang Y.H. (2014). A high-energy-density sugar biobattery based on a synthetic enzymatic pathway. Nat. Commun..

[6] Chang J.W., Wang C.G., Huang C.Y., Tsai T.D., Guo T.F., Wen T.C. (2011). Chicken albumen dielectrics in organic field-effect transistors. Adv. Mater..

[7] Yukimoto T., Uemura S., Kamata T., Nakamura K., Kobayashi N. (2011). Non-volatile transistor memory fabricated using DNA and eliminating influence of mobile ions on electric properties. J. Mater. Chem..

[8] Waser R., Aono M. (2007). Nanoionics-based resistive switching memories. Nat. Mater..

[9] Waser R., Dittmann R., Staikov G., Szot K. (2009). Redox-Based Resistive Switching Memories—Nanoionic Mechanisms, Prospects, and Challenges. Adv. Mater..

[10] Kwon D.H., Kim K.M., Jang J.H., Jeon J.M., Lee M.H., Kim G.H., Li X.S., Park G.S., Lee B., Han S. (2010). Atomic structure of conducting nanofilaments in TiO2 resistive switching memory. Nat. Nanotechnol..

[11] Wang X.F., Tian H., Zhao H.M., Zhang T.Y., Mao W.Q., Qiao Y.C., Pang Y., Li Y.X., Yang Y., Ren T.L. (2018). Interface Engineering with MoS2 -Pd Nanoparticles Hybrid Structure for a Low Voltage Resistive Switching Memory. Small.

[12] Yang Y., Gao P., Gaba S., Chang T., Pan X., Lu W. (2012). Observation of conducting filament growth in nanoscale resistive memories. Nat. Commun..

[13] Park J., Choi J., Chung D., Kim S. (2022). Transformed Filaments by Oxygen Plasma Treatment and Improved Resistance State. Nanomaterials.

[14] Shen Z., Zhao C., Qi Y., Xu W., Liu Y., Mitrovic I.Z., Yang L., Zhao C. (2020). Advances of RRAM Devices: Resistive Switching Mechanisms, Materials and Bionic Synaptic Application. Nanomaterials.

[15] Raeis-Hosseini N., Lee J.S. (2016). Controlling the Resistive Switching Behavior in Starch-Based Flexible Biomemristors. ACS Appl. Mater. Int..

[16] Hosseini N.R., Lee J.S. (2015). Resistive Switching Memory Based on Bioinspired Natural Solid Polymer Electrolytes. ACS Nano.

[17] Ko Y., Kim Y., Baek H., Cho J. (2011). Electrically Bistable Properties of Layer-by-Layer Assembled Multilayers Based on Protein Nanoparticles. ACS Nano.

[18] Zhang C., Shang J., Xue W., Tan H., Pan L., Yang X., Guo S., Hao J., Liu G., Li R.W. (2016). Convertible resistive switching characteristics between memory switching and threshold switching in a single ferritin-based memristor. Chem. Commun..

[19] Wang Y., Yan X., Dong R. (2014). Organic memristive devices based on silver nanoparticles and DNA. Org. Electron..

[20] Abbas Y., Dugasani S.R., Raza M.T., Jeon Y.R., Park S.H., Choi C. (2019). The observation of resistive switching characteristics using transparent and biocompatible Cu(2+)-doped salmon DNA composite thin film. Nanotechnology.

[21] Xing Y., Shi C.Y., Zhao J.H., Qiu W., Lin N.B., Wang J.J., Yan X.B., Yu W.D., Liu X.Y. (2017). Mesoscopic-Functionalization of Silk Fibroin with Gold Nanoclusters Mediated by Keratin and Bioinspired Silk Synapse. Small.

[22] Shi C., Wang J., Sushko M.L., Qiu W., Yan X., Liu X.Y. (2019). Silk Flexible Electronics: From Bombyx mori Silk Ag Nanoclusters Hybrid Materials to Mesoscopic Memristors and Synaptic Emulators. Adv. Funct. Mater..

[23] Chang Y.-C., Jian J.-C., Hsu Y.L., Huang W.-Y., Young S.-J. (2020). A Green Strategy for Developing a Self-Healing Gelatin Resistive Memory Device. ACS Appl. Polym. Mater..

[24] Chang Y.C., Wang Y.H. (2014). Resistive switching behavior in gelatin thin films for nonvolatile memory application. ACS Appl. Mater. Int..

[25] Ge L., Xuan W., Liu S., Huang S., Wang X., Dong S., Jin H., Luo J. (2018). Biomaterial Gelatin Film Based Crossbar Structure Resistive Switching Devices. IEEE Trans. Nanotechnol..

[26] Liu D., Jing Q., Cheng H. (2019). Synaptic-functional and fully water-soluble transient memristor made from materials compatible with semiconductor technology. Jpn. J. Appl. Phys..

[27] Koduvayur Ganeshan S., Selamneni V., Sahatiya P. (2020). Water dissolvable MoS2 quantum dots/PVA film as an active material for destructible memristors. New J. Chem..

[28] Guo Y., Hu W., Zeng F., Zhang C., Peng Y., Guo Y. (2020). Ultrafast degradable resistive switching memory based on α-lactose thin films. Org. Electron..

[29] Lin N., Hu F., Sun Y., Wu C., Xu H., Liu X.Y. (2014). Construction of White-Light-Emitting Silk Protein Hybrid Films by Molecular Recognized Assembly among Hierarchical Structures. Adv. Funct. Mater..

[30] Bae H., Lee B.H., Lee D., Seol M.L., Kim D., Han J.W., Kim C.K., Jeon S.B., Ahn D., Park S.J. (2016). Physically Transient Memory on a Rapidly Dissoluble Paper for Security Application. Sci. Rep..

[31] Ji X., Song L., Zhong S., Jiang Y., Lim K.G., Wang C., Zhao R. (2018). Biodegradable and Flexible Resistive Memory for Transient Electronics. J. Phys. Chem..

[32] Huang W.-Y., Chang Y.-C., Sie Y.-F., Yu C.-R., Wu C.-Y., Hsu Y.-L. (2021). Bio-Cellulose Substrate for Fabricating Fully Biodegradable Resistive Random Access Devices. ACS Appl. Polym. Mater..

[33] Chen Y.-C., Su Y.-K., Huang C.-Y., Yu H.-C., Cheng C.-Y., Chang T.-H. (2011). Bistable Resistive Switching Characteristics of Poly(2-hydroxyethyl methacrylate) Thin Film Memory Devices. Appl. Phys. Express.

[34] Zhang J., Yang H., Zhang Q.-l., Dong S., Luo J.K. (2013). Bipolar resistive switching characteristics of low temperature grown ZnO thin films by plasma-enhanced atomic layer deposition. Appl. Phys. Lett..

[35] Zhuge F., Dai W., He C.L., Wang A.Y., Liu Y.W., Li M., Wu Y.H., Cui P., Li R.-W. (2010). Nonvolatile resistive switching memory based on amorphous carbon. Appl. Phys. Lett..

